# Early Selection Enabled by the Implementation of Genomic Selection in *Coffea arabica* Breeding

**DOI:** 10.3389/fpls.2018.01934

**Published:** 2019-01-08

**Authors:** Tiago Vieira Sousa, Eveline Teixeira Caixeta, Emilly Ruas Alkimim, Antonio Carlos Baião Oliveira, Antonio Alves Pereira, Ney Sussumu Sakiyama, Laércio Zambolim, Marcos Deon Vilela Resende

**Affiliations:** ^1^BIOAGRO, BioCafé, Universidade Federal de Viçosa, Viçosa, Brazil; ^2^Empresa Brasileira de Pesquisa Agropecuária–Embrapa Café, BIOAGRO, BioCafé, Universidade Federal de Viçosa, Viçosa, Brazil; ^3^Universidade Federal do Triângulo Mineiro, Iturama, Brazil; ^4^Empresa Brasileira de Pesquisa Agropecuária–Embrapa Café, Viçosa, Brazil; ^5^Empresa de Pesquisa Agropecuária de Minas Gerais–Epamig, Viçosa, Brazil; ^6^Departamento de Fitotecnia, Universidade Federal de Viçosa, Viçosa, Brazil; ^7^Departamento de Fitopatologia, Universidade Federal de Viçosa, Viçosa, Brazil; ^8^Empresa Brasileira de Pesquisa Agropecuária–Embrapa Florestas, Viçosa, Brazil

**Keywords:** genetic gains, selective efficiency, genomic-enabled prediction accuracy, plant breeding, SNP molecular marker, complex traits, accelerating improvement

## Abstract

Genomic Selection (GS) has allowed the maximization of genetic gains per unit time in several annual and perennial plant species. However, no GS studies have addressed *Coffea arabica*, the most economically important species of the genus *Coffea*. Therefore, this study aimed (i) to evaluate the applicability and accuracy of GS in the prediction of the genomic estimated breeding value (GEBV); (ii) to estimate the genetic parameters; and (iii) to evaluate the time reduction of the selection cycle by GS in Arabica coffee breeding. A total of 195 Arabica coffee individuals, belonging to 13 families in generation of F_2_, susceptible backcross and resistant backcross, were phenotyped for 18 agronomic traits, and genotyped with 21,211 SNP molecular markers. Phenotypic data, measured in 2014, 2015, and 2016, were analyzed by mixed models. GS analyses were performed by the G-BLUP method, using the RKHS (Reproducing Kernel Hilbert Spaces) procedure, with a Bayesian algorithm. Heritabilities and selective accuracies were estimated, revealing moderate to high magnitude for most of the traits evaluated. Results of GS analyses showed the possibility of reducing the cycle time by 50%, maximizing selection gains per unit time. The effect of marker density on GS analyses was evaluated. Genomic selection proved to be promising for *C. arabica* breeding. The agronomic traits presented high complexity for they are controlled by several QTL and showed low genomic heritabilities, evidencing the need to incorporate genomic selection methodologies to the breeding programs of this species.

## Introduction

Genetic plant breeding started with the phenotypic selection of individuals that positively stood out in the segregating populations. In the 1980s, molecular markers were developed and used as an auxiliary tool to phenotypic information (Soller and Beckmann, 1983). With the evolution of molecular biology, in the 1990s, the Molecular Marker Assisted Selection (MAS) was proposed (Lande and Thompson, 1990), which enabled selecting individuals with specific alleles. However, MAS has shown to be inefficient in polygenic and/or low heritability traits (Bernardo, 2008). This limitation is mainly because molecular markers, on significant associations with QTL (Quantitative Trait Loci), are unable to capture genes of lesser effect (Hayes et al., 2009; Heffner et al., 2009; Xu et al., 2012).

Due to its potential and importance, genome-wide selection (GS) was developed by Meuwissen et al. (2001), being currently used in animal and plant studies (Crossa et al., 2010; de los Campos et al., 2010; Heffner et al., 2010; Jannink et al., 2010; Ornella et al., 2012; Azevedo Peixoto et al., 2017). The rapid adoption of this selective technique is due, among other factors, to the combination of expressive numbers of molecular markers, widely distributed throughout the species genome, and robust and accurate statistical methodologies. Therefore, the genetic value of individuals can be estimated (Longin et al., 2015), which allows increasing selection gain per unit time (Heffner et al., 2010). Several studies have demonstrated the high selective accuracy of GS [Bernardo and Yu, 2007; Wong and Bernardo, 2008; Heffner et al., 2009; Crossa et al., 2010; Davey et al., 2011; Garcia et al., 2011; Grattapaglia and de Resende, 2011; (Iwata et al., 2011; Resende et al., 2012b,c; de los Campos et al., 2013; Gianola, 2013)]. Moreover, GS has been reported as efficient for polygenic traits and traits with low heritability, high evaluation cost, and of difficult measurement (Heslot et al., 2015; Poland, 2015).

With the development of NGS (Next Generation Sequencing) platforms, GS has become a reality for several economically important species, including annual and perennial plants. The use of the NGS platforms has made SNP markers (Single Nucleotide Polymorphisms) economically feasible (Patel et al., 2015). SNP is the most abundant genetic variation in the genome (Kwok and Gu, 1999; Ganal et al., 2009) and allows the identification of polymorphism distributed throughout the species genome.

The use of SNP molecular markers in GS studies has been shown to be advantageous for several species. However, the procedure requires special care for polyploid species, which have subgenomes with duplicate regions or with high similarity, such as *Coffea arabica* species. These species originate from the natural cross from non-reduced gametes between the diploid species *Coffea canephora* and *Coffea eugenioides* (Lashermes et al., 1999), whose genomes have highly similar regions (Cenci et al., 2012). Although *C. arabica* is a true allotetraploid (Clarindo and Carvalho, 2008), its meiotic behavior is similar to that of a diploid with the bivalent formation (Lashermes et al., 2016). Thus, if the polymorphism detected by the SNP occurs between these regions of the sub genomes, this marker will not explain the phenotypic variation observed between individuals, being not informative (false SNP) (Vidal et al., 2010). Therefore, this SNP must be eliminated from the data set (Sant'Ana et al., 2018). Moreover, the objective must be to achieve the optimal number of molecular markers used to predict the genetic value of individuals. Excess markers associated with reduced number of observations (genotypes) can lead to multicollinearity problems. Thus, the analyses must use an optimal set of informative SNPs, maximizing the predictive accuracy estimates.

GS has an essential role in perennial plants (Resende et al., 2012a; Azevedo Peixoto et al., 2017). Despite the economic importance of *C. arabica*, no GS work has addressed this species. Coffee trees have been selected based on biometric analyses that use mainly phenotypic data of yield and resistance to diseases. Experiments with perennial species, such as *C. arabica*, usually present unbalanced data due to adversities in the field over time. Therefore, the use of the mixed models methodology, Residual or Restricted Maximum Likelihood/Best Linear Unbiased Prediction (REML/BLUP) (Patterson and Thompson, 1971; Henderson, 1975) has allowed, from phenotypic information, the accurate, and unbiased prediction of genetic values of individuals (Resende and Thompson, 2004; Viana et al., 2011; Barbosa et al., 2012; Ferreira et al., 2012; Pereira et al., 2013; Corrêa et al., 2015; Spinelli et al., 2015). For coffee, genetic gains have also been reported using molecular markers in studies on genetic diversity (Sousa et al., 2017), genetic maps (Pestana et al., 2015; Moncada et al., 2016), and assisted selection (Alkimim et al., 2017; Favoretto et al., 2017). However, due to the complexity and number of genes that control most of the agronomic traits of this species, GS studies are promising for they allow estimating the effects of all loci that explain the genetic variation (Heffner et al., 2009) and the genomic estimated breeding value (GEBV) (Meuwissen et al., 2001).

Given the above, this study aimed (i) to evaluate the applicability and accuracy of GS in the prediction of the GEBV; (ii) to estimate the genetic parameters; and (iii) to evaluate the time reduction of the selective cycle by GS in an Arabica coffee breeding.

## Materials and Methods

### Experimental Conduction

In the experimental area, soil liming and planting fertilization were performed according to the crop requirement. The genotypes were planted on February 11, 2011. Plants were arranged at spacing of 3.0 m between rows and 0.7 m between plants. No phytosanitary control method was used against rust, cercosporiosis, and leaf miner. The experiment was evaluated in the experimental area of the Department of Plant Pathology of the Universidade Federal de Viçosa, Brazil (lat. 20°44′25" S, long. 42°50′52" W), in 2014, 2015, and 2016.

### Genetic Material

**Figure 1:**
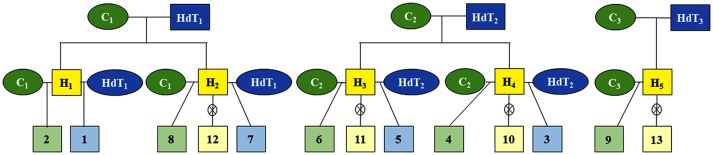
Heredogram of the 13 progenies of *Coffea arabica* from crosses between parents of the Catuaí group and Híbrido de Timor (HdT); C_1_, C_2_, and C_3_, genotypes Catuaí amarelo IAC 30, IAC 86, and IAC 64, respectively; HdT_1_, HdT_2_, and HdT_3_, genotypes Híbrido de Timor UFV 445-46, UFV 440-10, and UFV 530, respectively; H_1_, H_2_, H_3_, H_4_, and H_5_, hybrids from crosses between the parents Catuaí amarelo and Híbrido de Timor; 1, 3, 5, and 7, progenies of first rust-resistant backcross generation; 2, 4, 6, 8, and 9, progenies of first rust-resistance backcross generation; 10, 11, 12, and 13, progenies in the F_2_ generation.

From the cross between three parents of the Catuaí group and three parents of Híbrido de Timor (HdT), which contrast in relation to resistance to coffee rust, 13 progenies were obtained from the *C. arabica* breeding program of Epamig/UFV/Embrapa (Figure 1). These progenies are resistant backcrosses (BCr), susceptible backcrosses (BCs), and F_2_ (Figure 1 and Table 1) generations. In each progeny, 15 genotypes (repetitions) were analyzed, totaling 195 individuals.

**Table 1:** *Coffea arabica* progenies evaluated in 2014, 2015, and 2016 in Viçosa (Brazil).

**Progeny**	**Genotypes**	**Parent 1**	**Parent 2**
BCr 1	1–15	H 419-1 c-17	UFV 445-46
BCs 2	16–30	H 419-1 c-17	UFV 2143-235
BCr 3	31–45	H 514-8 c-387	UFV 440-10
BCs 4	46–60	H 514-8 c-387	UFV 2154-344
BCr 5	61–75	H 514-7 c-364	UFV 440-10
BCs 6	76–90	H 514-7 c-364	UFV 2154-344
BCr 7	91–105	H 419-10 c-214	UFV 445-46
BCs 8	106–120	H 419-10 c-214	UFV 2143-235
BCs 9	121–135	UFV 2148-57	H 513-5 c-14
F_2_ 10	136–150	H 514-8 c-387	–
F_2_ 11	151–165	H 514-7 c-364	–
F_2_ 12	166–180	H 419-10 c-214	–
F_2_ 13	181–195	H 513-5 c-14	–

*BCr, first resistant backcross; BCs, first susceptible backcross; F_2_, generation obtained by the selfing of F_1_ hybrids*.

### Phenotypic Evaluations

The phenotypic evaluations of 18 agronomic traits (11 continuous and seven categorical traits) were performed (Table 2) in the 195 *C. arabica* genotypes listed in Table 1, in 2014, 2015, and 2016.

**Table 2:** Phenotypic traits evaluated in 2014, 2015, and 2016 in Viçosa (MG).

**Traits**	**Type of evaluation**	
Yield	(Y)	Liters of fresh cherries harvested per plant
Leaf length (cm)	(LL)	Measured in the leaf of the third or fourth pair of a plagiotropic branch of the middle third of the plant (cm)
Leaf width (cm)	(LW)	
Branch length (cm)	(BL)	Measured in the plagiotropic branch of the middle third of the plant
Number of reproductive nodes	(NRN)	
Number of vegetative nodes	(NVN)	
Total number of fruits	(NF)	
Fruit volume	(FV)	
Plant height (cm)	(PH)	Measured in the orthotropic branch (from the soil surface to the final branch growth point)
Canopy diameter (cm)	(CD)	Measured transversely to the planting row, considering the greatest canopy longest
Stem diameter (cm)	(SD)	Measured at the stem region of the plant (about 5 cm from the soil surface)
Ripening fruit size	(RFS)	Evaluated by a score scale ranging from 1 to 3
Maturation uniformity	(MU)	Evaluated by a score scale ranging from 1 to 4
Maturation cycle	(MC)	Evaluated by a score scale ranging from 1 to 5
Rust incidence	(Rus)	
Cercosporiosis incidence	(Cer)	
Leaf miner infestation	(LM)	
Vegetative vigor	(Vig)	Evaluated by a score scale ranging from 1 to 10

The continuous traits were measured as described in Table 2. The categorical traits were evaluated by score scales. Ripening fruit size was evaluated by a score scale ranging from 1 to 3 (1: small; 2: medium; and 3: large fruits). Maturation uniformity was evaluated by a score scale ranging from 1 to 4 (1: uniform; 2: semi-uniform; 3: semi non-uniform; and 4: non-uniform maturation). Maturation cycle was evaluated by a score scale ranging from 1 to 5 (1: early; 2: semi-early; 3: intermediate; 4: semi-late; 5: late cycle). The incidence of coffee rust, cercosporiosis, and leaf miner was evaluated using a score scale ranging from 1 to 5, in which 1 corresponded to genotypes without symptoms and 5 referred to highly susceptible genotypes. Vegetative vigor was evaluated by a score scale ranging from 1 to 10, in which 1 was attributed to fully depauperate (depleted) plants and 10 was assigned to plants with maximum vegetative vigor.

### Genetic Parameters From Phenotypic Data

Thirteen progenies, which were composed of 15 plants (repetitions), totaling 195 genotypes were evaluated. Phenotypic data were corrected for years, plots, and years × plots interactions, from which the selective accuracies (r_yy_) and phenotypic heritabilities (${\text{h}}_{\text{phen}}^{2}$) of the 18 agronomic traits were estimated. Analyses were performed considering the linear mixed models (REML/BLUP procedure), implemented in the Selegen-REML/BLUP software (Resende, 2016). Genetic parameters were estimated by the individual analysis of the 18 traits, using the following statistical model:

$$\begin{array}{l} y=Xu+Zg+Wp+Vr+Tb+Ri+e \end{array}$$

Where:

*y* is the data vector;

*u* is the vector of the overall mean in each evaluation year;

*g* is the vector of progeny effects (random effect);

*p* is the permanent effects between plants (random effect);

*r* is the effects between population types (random effect);

*b* is the effects between plot (random effect);

*i* is the effects of progenies x years interaction (random effect);

*e* is the residue vector (random effect).

The uppercase letters represent the incidence matrices for these effects.

### Genomic DNA Extraction

Young and fully expanded leaves of the 195 genotypes were collected, and the genomic DNA was extracted using the methodology described by Diniz et al. (2005). The DNA concentration was verified in the NanoDrop 2000, and its quality was evaluated in 1% agarose gel.

The DNA concentration of the samples was standardized and sent to RAPiD GENOMICS, Florida/USA, for probes construction, sequencing, and identification of SNP molecular markers (Sousa et al., 2017).

### Quality Control of Molecular Markers

From 40,000 probes, 10,000 polymorphic probes were selected, and 21,211 SNP molecular markers were identified. Details on probes construction and SNPs identification can be obtained from Sousa et al. (2017). The SNP set was subject to quality analysis implemented in the Rbio software (Bhering, 2017). The quality parameters used were CR (Call Rate) and MAF (Minor Allele Frequency) equal to or higher than 90 and 5%, respectively. The critical level for MAF was obtained by the equation $MAF=\;\frac{1}{\sqrt{2N}}$, where N refers to the number of individuals evaluated. Moreover, to avoid the occurrence of false SNPs (Vidal et al., 2010) resulting from the polyploidy of *C. arabica*, SNPs that had the same genotype in all individuals, even when polymorphic, were eliminated. Thus, SNPs without genetic variance among the individuals that make up the study population were eliminated from the analysis.

### Cross-Validation

Cross-validation is a method used to evaluate the generalization capacity of a predictive model from a dataset. When applying this method, the dataset is partitioned into mutually exclusive subsets. The population, composed of 195 coffee trees, was divided into 13 folds−180 individuals were used for training or estimation of the predictive models and 15 individuals were used for validation. The process was repeated 13 times so that each part was used once as a validation set. In the end, the predictive capacity (r_gy_) of the GS model obtained by the result of the mean correlation between the GEBV and the observed phenotypic values was estimated.

### Genomic Selection

Genomic selection (GS) analyses were performed using the G-BLUP method via the RKHS (Reproducing Kernel Hilbert Spaces) procedure, with the Bayesian algorithm (Gianola, 2006). The BGLR (Bayesian Generalized Linear Regression) package (Perez and de los Campos, 2014), implemented in the software R (R Core Team, 2017), was used.

The general mixed linear model (Resende, 2007, 2008) was adjusted to estimate the effects of markers, according to the expression y = Xb + Wm + e, where y is the vector of phenotypic observations; b is the vector of fixed effects; m is the vector of random effects of markers; and e is the vector of random residue. Uppercase letters represent the incidence matrices for these effects. The incidence matrix X contains the values 0, 1, and 2 for the number of alleles of the marker (or the so-called QTL) in a diploid individual. The genomic mixed model equations for the prediction of m via the G-BLUP method are equivalent to:

$$\begin{array}{l} \left[\begin{array}{ll} X\prime X & X\prime W\\ W\prime X & W\prime W+I\frac{\sigma_{e}^{2}}{\left(\sigma_{g}^{2}/n_{Q}\right)} \end{array}\right]\left[\begin{array}{l} \hat{b}\\ \hat{m} \end{array}\right]=\left[\begin{array}{c} X\prime_{y}\\ W\prime_{y} \end{array}\right] \end{array}$$

The genomic estimated breeding value (GEBV) of individual j is given by $GEBV=\underset{i}{\sum }w_{ij}{\hat{m}}_{i}$, in which W_i_ is equal to 0, 1, or 2 for the genotypes mm, Mm, and MM, respectively, for the biallelic and codominant marker i (SNP); and W_ij_ is the element i of row j of matrix W, regarding individual j.

### Predictive Capacity and Accuracy of GS

The predictive capacity (*r*_*gy*_) is estimated by correlating the predicted genomic values with the corrected phenotypic values, being equivalent to the predictive capacity of the GS to estimate phenotypes (Resende et al., 2014a).

The accuracy was obtained by the estimator $r_{gg\;}=\frac{r_{gy}}{\sqrt{h^{2}}}$, in which *r*_*gy*_ is the predictive capacity of the GS, and *h*^2^ is the individual heritability (Legarra et al., 2008).

### Number of QTL and Individuals

The number of QTL (nQTL) controlling each trait was estimated by the expression $n_{QTL}=\frac{\left(1-r_{gg}^{2}\right)Nh^{2}}{r_{gg}^{2}}$, where *r*_*gg*_is the accuracy of the GS; N is the number of individuals in the population; and *h*^2^ in the individual heritability (Resende et al., 2008). The individual heritability was estimated by: $h^{2}=\sigma_{g}^{2}/\left(\sigma_{g}^{2}+\sigma_{p}^{2}+\sigma_{r}^{2}+\sigma_{b}^{2}+\sigma_{i}^{2}+\sigma_{e}^{2}\right)$, where $\sigma_{i}^{2}$ is the variance component associated to the i effect.

The number of individuals (Ni) that must be evaluated to obtain the desired accuracy was estimated by the expression $Ni=\frac{r_{gg\;}^{2}n_{QTL}}{\left(1-r_{gg}^{2}\right)h^{2}}$, in which *r*_*gg*_is the accuracy of the GS; *n*_*QTL*_ is the number of QTL controlling each trait; and *h*^2^ is the individual heritability (Resende et al., 2014a).

### Markers Density

The effect of the number of markers on the selective accuracy was evaluated. Predictive accuracy, with a set of markers composed of different SNP densities, was estimated by the G-BLUP method, using the RKHS (Reproducing Kernel Hilbert Spaces) procedure with a Bayesian algorithm (Gianola, 2006). The BGLR package (Perez and de los Campos, 2014), implemented in the software R (R Core Team, 2017), was used. These analyses were performed with a set of markers composed of 1,000; 4,000; 8,000; 12,000; 16,000; 20,000; and 20,477 SNPs selected to representatively sample the original data set. Cross-validation was performed using 13 folds.

### Selective Efficiency of GS

The selective efficiency of GS (Ef), compared with selection based on phenotypes alone, was estimated by the expression $Ef=\frac{r_{gg}L_{f}}{r_{yy}L_{GS}}$, in which r_gg_ is the selective accuracy of GS; L_f_ is the mean time required for the selection cycle based on phenotypes; r_yy_ is the accuracy of the phenotypic selection; L_GS_ is the mean time required for the selection cycle based on GS (Resende et al., 2012d). Efficiency analyses were estimated considering 24 years to obtain phenotypic accuracies, according to the mean release time of an Arabica coffee cultivar composed of four selection cycles, each cycle lasting 6 years. Conversely, the selective accuracies of GS were estimated considering 12 and 24 years. This 12 year period is the minimum duration for the use of SNPs, considering four selection cycles, each one totaling 3 years. Although the application of SNP allows for the selection at the seed stage, a 3 year cycle was considered since this is the period required for the coffee trees to reproduce.

## Results

### Genetic Parameters From Phenotypic Data

Eighteen traits of agronomic importance were analyzed in 195 coffee trees. The individuals make up 13 families, which were obtained from crosses between parents of the Catuaí group and Híbrido de Timor (HdT). From the phenotypic data, heritabilities (h^2^_phen_) and selective accuracies (r_yy_) were estimated using the mixed model methodology (REML/BLUP) (Table 3). Stem diameter (SD) had the lowest estimate of h^2^_phen_ (0.01); conversely, plant height (PH) and canopy diameter (CD) showed the highest values for this parameter (0.90). Most of the evaluated traits presented high magnitude of r_yy_, with the exception of SD.

**Table 3:** Estimate of genetic parameters obtained by mixed model analyses (REML/BLUP), results of the Genome-wide Selection (GS), and estimates of the number of individuals to obtain a desired selective accuracy (Ni) for 18 morpho-agronomic traits in a *Coffea arabica* breeding population evaluated in 2014, 2015, and 2016.

	**REML/BLUP**		**Genomic selection (GS)**								**Number of individuals (Ni)**				
**Trait**	**${\text{h}}_{\text{phen}}^{2}$**	**r_**yy**_**	**${\text{h}}_{\text{a}}^{2}$**	**sd_**h**_**	**r_**gy**_**	**sd_**r**_**	**b**	**sd_**b**_**	**r_**gg**_**	**n_**QTL**_**	**r_**ggd**_ 0.5**	**r_**ggd**_ 0.6**	**r_**ggd**_ 0.7**	**r_**ggd**_ 0.8**	**r_**ggd**_ 0.9**
Y	0.55	0.74	0.26	0.03	0.13	0.27	1.63	3.01	0.25	751	964	1,626	2,778	5,140	12,326
LL	0.42	0.65	0.29	0.02	0.06	0.23	0.87	2.21	0.12	3,981	4,530	7,644	13,057	24,160	57,935
LW	0.44	0.66	0.32	0.05	0.03	0.25	0.44	2.62	0.06	17,758	18,631	31,440	53,701	99,365	238,281
BL	0.78	0.88	0.41	0.04	0.32	0.34	1.20	1.30	0.50	244	198	335	572	1,058	2,538
NRN	0.49	0.70	0.23	0.02	−0.01	0.21	0.25	3.25	–	–	–	–	–	–	–
NVN	0.44	0.66	0.46	0.04	0.38	0.21	1.92	1.63	0.56	199	143	242	413	765	1,834
NF	0.49	0.70	0.34	0.05	0.14	0.20	1.33	2.10	0.23	1,157	1,134	1,913	3,267	6,046	14,498
FV	0.57	0.76	0.25	0.03	0.11	0.17	1.23	1.90	0.21	1,081	1,418	2,393	4,087	7,562	18,133
PH	0.90	0.95	0.46	0.04	0.38	0.18	1.18	0.77	0.56	202	146	246	420	777	1,864
CD	0.90	0.95	0.45	0.03	0.40	0.22	1.46	0.90	0.61	149	112	189	322	596	1,429
SD	0.01	0.10	0.16	0.01	0.06	0.26	1.14	2.90	0.14	1,658	3,363	5,674	9,692	17,934	4,306
RFS	0.50	0.71	0.36	0.04	0.23	0.24	1.52	2.01	0.39	394	370	624	1,066	1,973	4,730
MU	0.30	0.55	0.28	0.03	0.03	0.27	0.62	3.13	0.06	14,775	17,841	30,107	51,424	95,152	228,177
MC	0.72	0.85	0.31	0.05	0.12	0.19	1.31	2.15	0.21	1,313	1,434	2,421	4,134	7,650	18,345
Rus	0.61	0.78	0.31	0.04	0.26	0.22	1.50	1.34	0.46	221	237	40	684	1,265	3,033
Cer	0.38	0.62	0.44	0.05	0.31	0.30	1.45	1.55	0.47	304	231	390	666	1,233	2,957
LM	0.30	0.55	0.30	0.04	0.18	0.24	1.34	1.71	0.33	476	536	904	1,544	2,858	6,852
Vig	0.70	0.84	0.34	0.04	0.21	0.19	1.38	1.39	0.36	440	437	738	1,260	2,332	5,592

*h2_phen_, heritability estimated from phenotypic data; r_yy_, accuracy of the selection obtained by the REML/BLUP method estimated from phenotypic data; ${\text{h}}_{\text{a}}^{2}$, genomic heritability; sd_h_, standard error of the ${\text{h}}_{\text{a}}^{2}$ estimates; r_gy_, predictive capacity of GS; sd_r_, standard error of the r_gy_ estimates; b, prediction bias; sd_b_, standard error of the b estimates; r_gg_, selective accuracy of GS; nQTL, estimate of the number of QTL controlling the trait; r_ggd_, desired selective accuracy; Ni, number of individuals evaluated to obtain a desired r_ggd_; Y, yield; LL, leaf length; LW, leaf width; BL, plagiotropic branch length; NRN, number of reproductive nodes; NVN, number of vegetative nodes; NF, number of fruits per plagiotropic branch; FV, fruits volume per plagiotropic branch; PH, plant height; CD, canopy diameter; SD, stem diameter; RFS, ripening fruits size; MU, maturation uniformity; MC, maturation cycle; rus, incidence of rust; Cer, Incidence of cercosporiosis; LM, leaf miner infestation; Vig, vegetative vigor*.

### Quality Control of Molecular Markers

**Figure 2:**
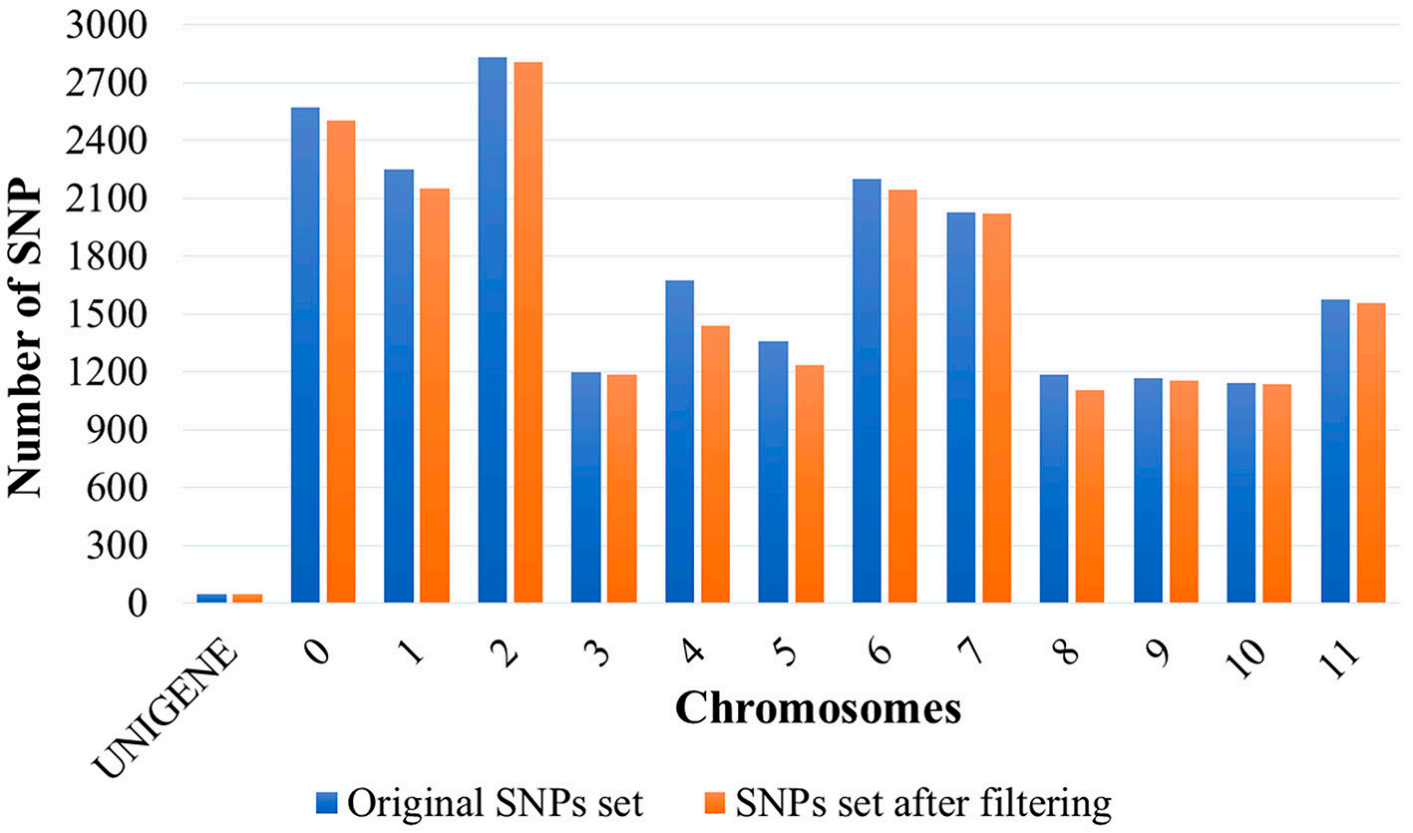
SNP molecular markers distributed throughout the UNIGENES from the EST sequences of *Coffea arabica* and the 11 chromosomes and the “chromosome 0” of *Coffea canephora*. “Chromosome 0” consists of a set of non-ordered sequence scaffolds (Denoeud et al., 2014).

Coffee trees, besides being phenotyped, were genotyped with 21,211 SNP markers. After quality analyses, 20,477 SNPs were selected. The initial set of SNP markers reduced by 3.46% (Figure 2). The most significant reduction (percentage) in the number of markers was observed on chromosome 4, corresponding to 14.21%. Markers were widely distributed, being identified on all chromosomes of coffee. The number of SNPs per chromosome ranged from 49 (UNIGENE) to 2,804 (chromosome 2), with a mean of 1,575 SNPs per chromosome.

### Genomic Heritability

Genomic heritabilities (${\text{h}}_{\text{a}}^{2}$) were estimated from the predictive equations of genomic selection. Estimates of ${\text{h}}_{\text{a}}^{2}$ ranged from 0.16, for stem diameter (SD), to 0.46, for number of vegetative nodes (NVN) and plant height (PH) (Table 3). For all the evaluated traits, ${\text{h}}_{\text{a}}^{2}$ estimates had a standard error equal to or lower than 0.05.

### Predictive Capacity and Prediction Bias

Estimates of the predictive capacity (r_gy_) of the 18 traits ranged from −0.01 to 0.40, for number of reproductive nodes (NRN) and canopy diameter (CD), respectively (Table 3). The standard error of the estimates ranged from 0.17 to 0.34. In addition to CD, the highest estimates of predictive capacity were observed for number of vegetative nodes (NVN) and plant height (PH). Results of ${\text{h}}_{\text{a}}^{2}$ and r_gy_ showed a high positive association, with a correlation coefficient of 88%. Prediction bias estimates (b) ranged from 0.25 to 1.92 for number of reproductive nodes (NRN) and number of vegetative nodes (NVN), respectively. Most of the traits evaluated showed a b estimate close to the unit. The standard error of these estimates ranged from 0.77 to 3.25.

### Selective Accuracy of GS

Selective accuracy estimates obtained with the GS (r_gg_) are presented in Table 3. r_gg_ was not estimated for number of reproductive nodes (NRN) since its predictive capacity estimate was negative. The estimated r_gg_ values of the other traits ranged from 0.06, for maturation uniformity (MU) and leaf width (LW), to 0.61, for canopy diameter (CD). A high correlation was observed between the estimates of r_gg_ and ${\text{h}}_{\text{a}}^{2}$ (82%) and between r_gg_ and r_gy_ (99%).

### Number of QTL

The number of QTL that controlled the trait (nQTL) ranged from 149 to 17,758 for canopy diameter (CD) and leaf width (LW), respectively (Table 3). The agronomic traits showed to be controlled by a large number of QTL. The nQTL estimated for grain yield and coffee rust incidence, which are the main traits in a coffee breeding program, were 751 and 221, respectively. These results showed an inversely proportional relationship between selective accuracy (r_gg_) and number of QTL.

### Number of Individuals to Obtain a Desired Selective Accuracy

The estimate of the number of individuals (*Ni*) required to obtain a desired selective accuracy (r_ggd_) is presented in Table 3. Results confirm the requirement of the evaluation of more individuals when high r_ggd_ estimates are intended. Based on the data, 322–53.701 of individuals should be evaluated for canopy diameter (CD) and leaf width (LW), respectively, to obtain a selective accuracy estimate of 0.7, considered as of high magnitude (Resende and Duarte, 2007). For most of the traits, more than 1,000 individuals must be evaluated to obtain r_ggd_ equal to 0.7.

### Markers Density

GS predictive analyses using different marker densities, in general, evidenced the increase in selective accuracy (r_gg_) when using a larger number of SNPs (Table 4). However, when the optimal number of markers was reached, which maximizes the r_gg_ estimates, selective accuracies decreased with the increase in the number of markers.

**Table 4:** Selective accuracy estimated from different densities of SNP markers and efficiency of genome-wide selection (GS) in relation to phenotypic selection in a *Coffea arabica* breeding population.

**TRAIT**	**Number of SNP^a^**							**Years for GS analysis^b^**	
	**1.000**	**4.000**	**8.000**	**12.000**	**16.000**	**20.000**	**20.477**	**12**	**24**
Y	-0.08	0.10	0.21	0.25	0.27	0.26	0.25	0.68	0.34
LL	0.14	0.18	0.22	0.07	0.12	0.16	0.12	0.37	0.18
LW	0.00	0.26	0.11	0.27	0.22	0.19	0.06	0.18	0.09
BL	0.42	0.44	0.50	0.51	0.51	0.56	0.50	1.13	0.56
NRN	0.03	0.04	−0.07	0.12	0.09	0.06	−0.01	–	–
NVN	0.44	0.33	0.43	0.44	0.46	0.50	0.56	1.68	0.84
NF	0.08	0.18	0.29	0.33	0.27	0.25	0.23	0.67	0.33
FV	0.23	0.27	0.30	0.24	0.21	0.19	0.21	0.55	0.28
PH	0.56	0.48	0.58	0.57	0.48	0.54	0.56	1.17	0.59
CD	0.37	0.48	0.52	0.58	0.59	0.57	0.61	1.28	0.64
SD	−0.03	0.14	0.16	−0.04	−0.02	−0.05	0.14	2.77	1.38
RFS	0.33	0.47	0.30	0.40	0.31	0.36	0.39	1.09	0.54
MU	0.07	0.15	0.17	0.09	0.01	0.25	0.06	0.22	0.11
MC	−0.07	0.11	0.01	0.27	0.10	0.18	0.21	0.49	0.25
Rus	0.19	0.42	0.40	0.24	0.24	0.38	0.46	1.18	0.59
Cer	0.39	0.37	0.35	0.36	0.41	0.44	0.47	1.52	0.76
LM	0.23	0.28	0.23	0.23	0.27	0.26	0.33	1.20	0.60
Vig	0.27	0.46	0.43	0.37	0.49	0.37	0.36	0.86	0.43

a *Selective accuracy estimated from different densities of SNP markers*;

b *Efficiency of genome-wide selection (GS) in relation to phenotypic selection*;

*Y, yield; LL, leaf length; LW, leaf width; BL, plagiotropic branch length; NRN, number of reproductive nodes; NVN, number of vegetative nodes; NF, number of fruits per plagiotropic branch; FV, fruits volume per plagiotropic branch; PH, plant height; CD, canopy diameter; SD, stem diameter; RFS, ripening fruits size; MU, Maturation uniformity; MC, Maturation cycle; Rus, Incidence of rust; Cer, Incidence of cercosporiosis; LM, Leaf miner infestation; Vig, Vegetative vigor*.

### Efficiency of GS

The efficiency of GS analysis in relation to phenotypic selection is presented in Table 4. The GS analysis was not performed for number of reproductive nodes (NRN) since the estimate its predictive capacity was close to zero (Table 3). Results demonstrated the possibility of reducing the cycle time by 50%. In nine traits, GS was more efficient than the phenotypic selection when reducing the selection cycle time from 24 to 12 years, including coffee rust incidence (Rus), cercosporiosis incidence (Cer), and leaf miner infestation (LM).

## Discussion

### Genetic Parameters From Phenotypic Data

Heritabilities (${\text{h}}_{\text{phen}}^{2}$) and selective accuracies (r_yy_) of 18 coffee trees agronomic traits were estimated from phenotypic data. The magnitude of the ${\text{h}}_{\text{phen}}^{2}$ estimates for most traits was considered as from intermediate to high. Heritability represents how much of the phenotypic variation is due to genetic influences (Krueger et al., 2008). Traits with lower heritability are usually controlled by more genes, and therefore, the selection is more complex. In general, the traits evaluated showed r_yy_ of high magnitude. Accuracy depends mainly on the ratio between the mean residual variation and the genotype variation. In its turn, the mean residual variation depends on the number of replications and the control when conducting the experiments (Resende and Duarte, 2007). Selective accuracy reflects the quality of the information and approaches used in genetic values prediction. This measure is associated with the precision of selection and refers to the correlation between predicted genetic values and true genetic values of individuals. The higher the selective accuracy in the evaluation of an individual, the higher is the evaluation confidence and genetic value predicted for the individual.

For non-normally distributed traits such as Ripening fruit size (evaluated by a score scale ranging from only 1 to 3) or Maturation uniformity (1–4), the technique called Generalized Linear Model should be used. This was done and the results did not differ so much from those got by using the standard procedure of Linear Mixed Model. This is in line with theory, which preconizes that the higher the number of score scale classes, the smaller the benefit from using the Generalized Linear Model technique. For small class numbers, the expected theoretical benefits are below 10%.

### Quality Control of Molecular Markers

The coffee trees belonging to breeding populations were genotyped. More than 20,000 SNPs were identified, which were widely distributed in the genome and all coffee chromosomes. This number of identified SNPs is higher than those that have been published so far. From expressed sequence tag (EST) of *C. arabica, C. canephora*, and *C. racemosa*, 7,538 SNPs were identified, and 180 were selected for validation in *C. arabica* and *C. canephora* accessions from Puerto Rico (Zhou et al., 2016). In another work, 952 SNPs were located on a genetic map of *C. arabica* (Moncada et al., 2016). From Ethiopian *C. arabica* collection and some Brazilian cultivars, 6,696 SNPs were identified and 2,587 with quality were selected for Genome-wide association studies (GWAS) (Sant'Ana et al., 2018).

### Genomic Heritability

From the information of the GS predictive equations, genomic heritability (${\text{h}}_{\text{a}}^{2}$) were estimated, showing low or moderate magnitudes and a standard error equal to or lower than 0.05. Traits with low heritability are expected to present lower predictive capacity (Legarra et al., 2008). Heritability estimate allows predicting the progress to be obtained with the selection. The lower the heritability of the trait, the more complex is the selection of traits, and consequently, the lower is the capacity to correctly predict phenotypes of individuals not sampled for model computation. This fact was demonstrated in simulations by Grattapaglia and de Resende (2011), who verified that the increase in the heritability of the trait leads to an increase in the accuracy of the GS.

### Predictive Capacity

The correlation coefficient or predictive capacity (r_gy_) and the regression coefficient or prediction bias (b), associated with observed phenotypic values and predicted genetic values, are practical measures of the ability of the methods to make accurate and unbiased predictions, respectively (Resende et al., 2014b). The results for ${\text{h}}_{\text{a}}^{2}$ and r_gy_ showed a high positive association, with a correlation coefficient of 88%. As observed in this work, the association between predictive capacity and heritability has been reported by other researchers (Cavalcanti et al., 2012; Gois et al., 2016). Prediction bias for most of the evaluated traits showed a b estimate close to the unit. This result indicates that the prediction was unbiased and therefore effective in predicting the true magnitudes of the differences between individuals (Resende et al., 2012a).

### Selective Accuracy of GS

The selective accuracy estimates of GS (r_gg_) were of low to moderate magnitude (Resende and Duarte, 2007). Selective accuracy (r_gg_) refers to the correlation between the true genotypic value of the genetic treatment and that estimated or predicted from the phenotypic information (Gois et al., 2016). The adequate r_gg_ values are close to the unit. The lower the absolute deviations between the parametric genetic values and the estimated or predicted genetic values, the higher is the accuracy (Resende and Duarte, 2007). The value of this measure indicates how accurate the model is in estimating the GEBV.

The low magnitudes of r_gg_ observed in some traits can be explained by the reduced population size and, mainly, by the effective population size. However, for being a perennial species with a high maintenance cost, an increase in the population size may hinder the breeding program. In studies with wheat populations, the increase in population size increased the selective accuracies estimates (Heffner et al., 2011a,b).

A high correlation was observed between the estimates of r_gg_ and ${\text{h}}_{\text{a}}^{2}$ (82%). A positive correlation between selective accuracy and heritability has also been reported for yellow rust and stem rust in wheat (Ornella et al., 2012).

The success of genomic selection is influenced by several factors, which consequently interfere with the selective accuracy of a GS model, such as the training population size, the actual population size, markers density, trait heritability, and number of QTL controlling the traits (Grattapaglia and de Resende, 2011; Desta and Ortiz, 2014). Among these factors, heritability and number of QTL controlling the trait are inherent to the genetic architecture of the trait (Resende et al., 2014b). Moreover, the genetic structure of the population may influence genomic predictions (Zhang et al., 2010; Li et al., 2014; Wang et al., 2014). In this sense, the different allelic frequencies between subpopulations can produce false associations between molecular and phenotypic data (Price et al., 2010) and thus overestimate heritability and reduce selective accuracy (Riedelsheimer et al., 2012; Wray et al., 2013).

### Number of QTL

The traits evaluated presented large numbers of QTL (nQTL). An inversely proportional relation was observed between nQTL and selective accuracy (r_gg_). This fact can be justified by the increase in the predictive complexity in function of the larger number of genes controlling the trait. When several genes affect a trait, their effects are usually small, and, consequently, the accurate estimation is challenging (Goddard, 2009). This phenomenon evidences the importance of using high-density SNP markers in the predictive analyses, aiming to identify SNP in linkage disequilibrium with all the QTL controlling the traits of interest. Studies with forest species (Grattapaglia and de Resende, 2011; Iwata et al., 2011) and maize (Riedelsheimer et al., 2012) revealed no relationship between the number of QTL and the phenotypic or genotypic accuracy.

### Number of Individuals to Obtain a Desired Selective Accuracy

Most of the analyzes traits required the evaluation of more than 1,000 individuals to obtain a selective accuracy of 0.7, considered by Resende and Duarte (2007) as of high magnitude. The larger the number of individuals genotyped, the more reliable estimates of the SNPs effects are obtained since each individual is a is a repetition.

### Markers Density

The results of the predictive analyses using different markers densities revealed the increase in the selective accuracy (r_gg_) with the increase in the number of SNPs. The increase in the markers density guarantees the conservation of marker-QTL associations and allows obtaining high selective accuracies (Desta and Ortiz, 2014). Marker density is determined primarily by the extent of the linkage disequilibrium (LD) and sample size. Therefore, if the number of markers used is reduced, the population size should be increased (Grattapaglia and de Resende, 2011). However, when the optimal number of markers was reached, which maximizes the r_gg_ estimates, the selective accuracy decreased with the increase in the number of markers. Results were similar to those of other researchers (Fernando et al., 2007; Cavalcanti et al., 2012), where the increase in the number of markers did not show a linear relationship with the accuracy of the GS. Studies with simulated data have demonstrated that the use of a large number of markers led to a reduction in the limitation imposed by the small size of the training population (Resende, 2008).

### Efficiency of the GS

The results of the efficiency of the GS in relation to phenotypic selection showed the possibility of reducing the selection cycle time by 50% for nine evaluated traits. This reduction allows the breeders to maximize the genetic gains per unit time, besides early selection (Asoro et al., 2013; Simeão Resende et al., 2014; Yabe et al., 2018). By applying this strategy, breeders will be able to eliminate undesirable genotypes and focus efforts on potential genotypes, and therefore reduce maintenance costs for breeding populations in the field. The fact that selection based on phenotypic data is more efficient than genomic selection for some traits can be explained by the number of evaluated genotypes.

Genomic selection uses much more information on parentage than phenotypic selection, which is based on pedigree. Then genomic heritability and accuracy of genomic selection can sometimes be higher than those parameters from phenotypic selection. And this can be explained by the many more genetic relationship in the G (the genomic relationship matrix) than in A (the genetic relationship matrix based on genealogy). This increase in the amount of information by using the genomic matrix G can, sometimes, lead to better and more precise estimations, and predictions. This fact can explain the differences between the results from genomic and phenotypic approaches observed in our paper. Another aspect is referring to the ability of SNPs to capture causal variants associated to the traits. Some markers are more informative for some traits than for others. This can explain the different behaviors presented by the different traits.

## Perspectives on the Use of GS in *Coffea arabica*

With globalization and a significant increase in the world's population, the demand for techniques to assist breeders in the development of new cultivars has intensified. In this sense, the elucidation and use of genomic information, including GS studies, allows the access to genetic information, which is potentially useful for coffee breeding programs. The increased knowledge of the genetic variation in breeding populations will reduce the time and resources intended to development a new cultivar. Moreover, it will enable the selection of breeding lines/cultivars of superior quality, which are more adapted and productive.

## Conclusion

Genome-wide selection proved to be promising for *C. arabica* breeding for reducing the selection cycle time. Agronomic traits are highly complex; they are controlled by several QTL, and present low genomic heritabilities, evidencing the need to incorporate genomic selection methodologies in the breeding programs of this species.

## Author Contributions

TS conceived the work, analyzed the data, discussed the different aspects of Genomic Selection, and wrote the first draft of the paper. EC conceived the work, supervised the data analysis, discussed the different aspects of Genomic Selection, and wrote the paper. EA provided technical assistance with DNA extraction, marker analysis, and wrote the paper. AO, AP, NS, and LZ provided phenotypic data from the breeding program and supervised the work. MR analyzed the data and discussed the different aspects of Genomic Selection, while the final manuscript was written in collaboration.

### Conflict of Interest Statement

The authors declare that the research was conducted in the absence of any commercial or financial relationships that could be construed as a potential conflict of interest.
